# Palliation of a Heterotaxy Single Ventricle Neonate with Pulmonary Atresia and Obstructed TAPVR

**DOI:** 10.1007/s00246-023-03101-4

**Published:** 2023-03-04

**Authors:** Justin Robinson, Joseph M. Forbess, Michael Slack, Julianne Moss, Alicia Chaves

**Affiliations:** 1 University of Maryland Heart and Vascular Center, Baltimore, MD USA; 2 Children’s Heart Program, University of Maryland School of Medicine, Baltimore, MD USA; 3 Baltimore, USA

**Keywords:** Congenital heart disease, TAPVR, Heterotaxy syndrome, Single ventricle, Palliation

## Abstract

Patients born with obstructed total anomalous pulmonary venous return have a high risk of morbidity and mortality in the neonatal period, which only increases when combined with single ventricle physiology and non-cardiac congenital anomalies such as heterotaxy syndrome. Despite advances in management of congenital heart disease, surgery within the first weeks of life to repair the pulmonary venous connection and establish pulmonary blood flow with a systemic-to-pulmonary shunt has historically led to disappointing outcomes. A multidisciplinary approach with pediatric interventional cardiology and cardiac surgery is required to reduce morbidity and mortality in this extremely high-risk patient population. Extending the time between birth and cardiac surgery can lessen postoperative complications and mortality risk, especially in patients with abnormal thoracoabdominal relationships. Our team was able to successfully utilize transcatheter stent placement in a vertical vein and patent ductus arteriosus to delay and stage cardiac surgeries in an infant born with obstructed total anomalous pulmonary venous return, unbalanced atrioventricular septal defect with pulmonary atresia and heterotaxy, thus reducing the morbidity and mortality associated with this diagnosis.

## Introduction

Heterotaxy is a deviation from the normal embryologic looping that determines the right to left sidedness and the relationship of organs in the thoracic and abdominal cavities to each other. Cardiac abnormalities associated with this condition include systemic and pulmonary venous return defects, atrioventricular valve anomalies, atrial and ventricular septal defects, pulmonary outflow tract obstruction, and abnormal ventriculoarterial connections [1]. Single ventricle (SV) physiology associated with pulmonary atresia (PA) or stenosis (PS), and total anomalous pulmonary venous return (TAPVR) is a common constellation of findings in this population. Patients with SV physiology and TAPVR have historically poor outcomes, and this is especially true for SV patients with obstructed TAPVR [2].

We present the case of an infant diagnosed with heterotaxy syndrome, SV physiology and obstructed TAPVR. To avoid the well-described risks of a single-stage neonatal surgical repair of TAPVR combined with a systemic-to-pulmonary arterial shunt procedure, we pursued a completely transcatheter strategy in the neonatal period both to relieve the pulmonary venous obstruction and provide a stable source of pulmonary blood flow.

## Case Report

The patient was born at 39 weeks via vaginal delivery with initial APGAR scores of 8 and 9, respectively. Radiologic imaging confirmed the prenatal findings of heterotaxy syndrome with levocardia, situs ambiguous, and asplenia. A postnatal echocardiogram showed a severely unbalanced (left-dominant) atrioventricular septal defect, PA, obstructed supracardiac TAPVR with a stenotic vertical vein, and a left-sided inferior vena cava (IVC).

**Fig. 1:**
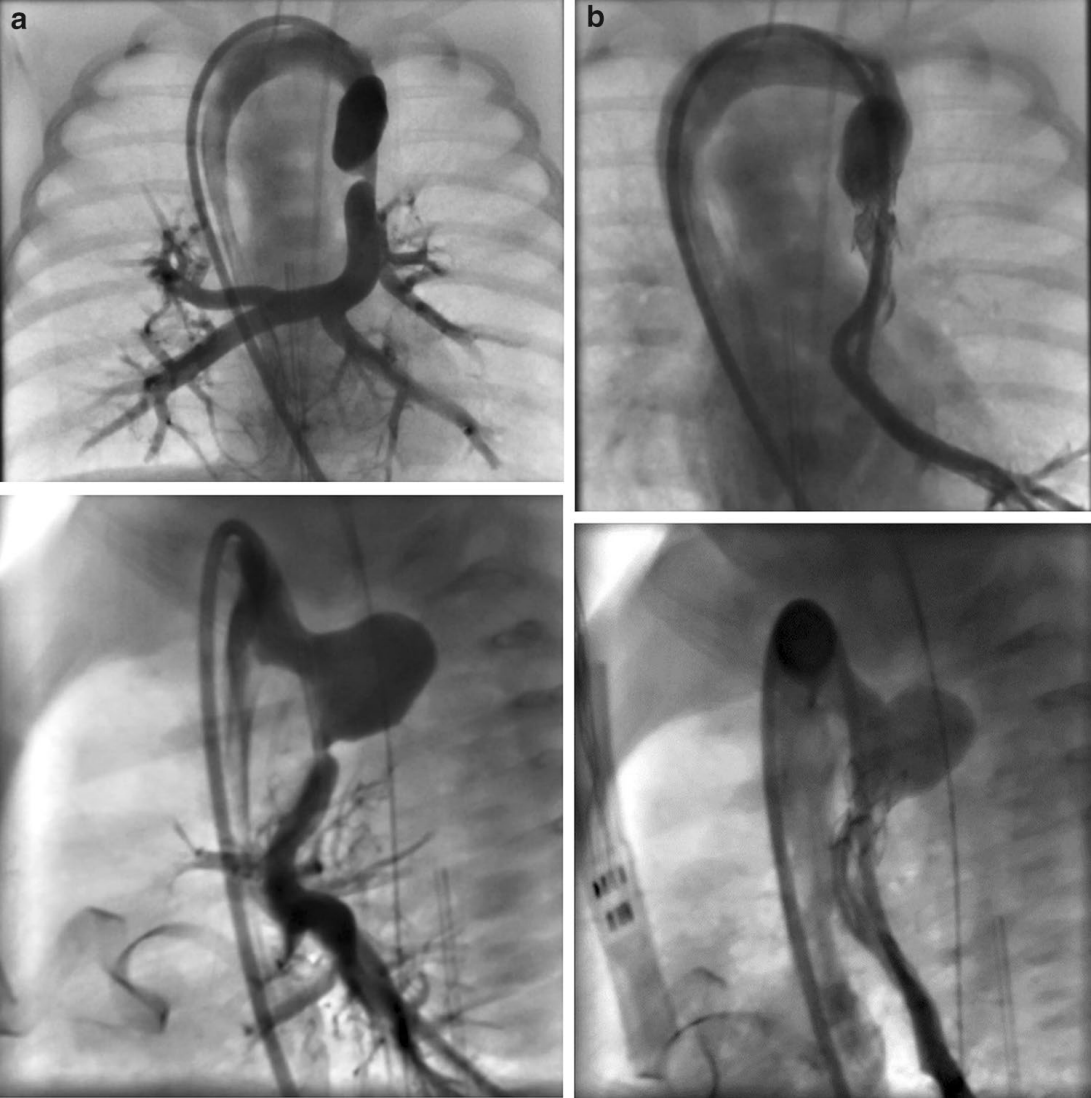
**a** pre-vertical vein stent, **b** post-vertical vein stent

**Fig. 2:**
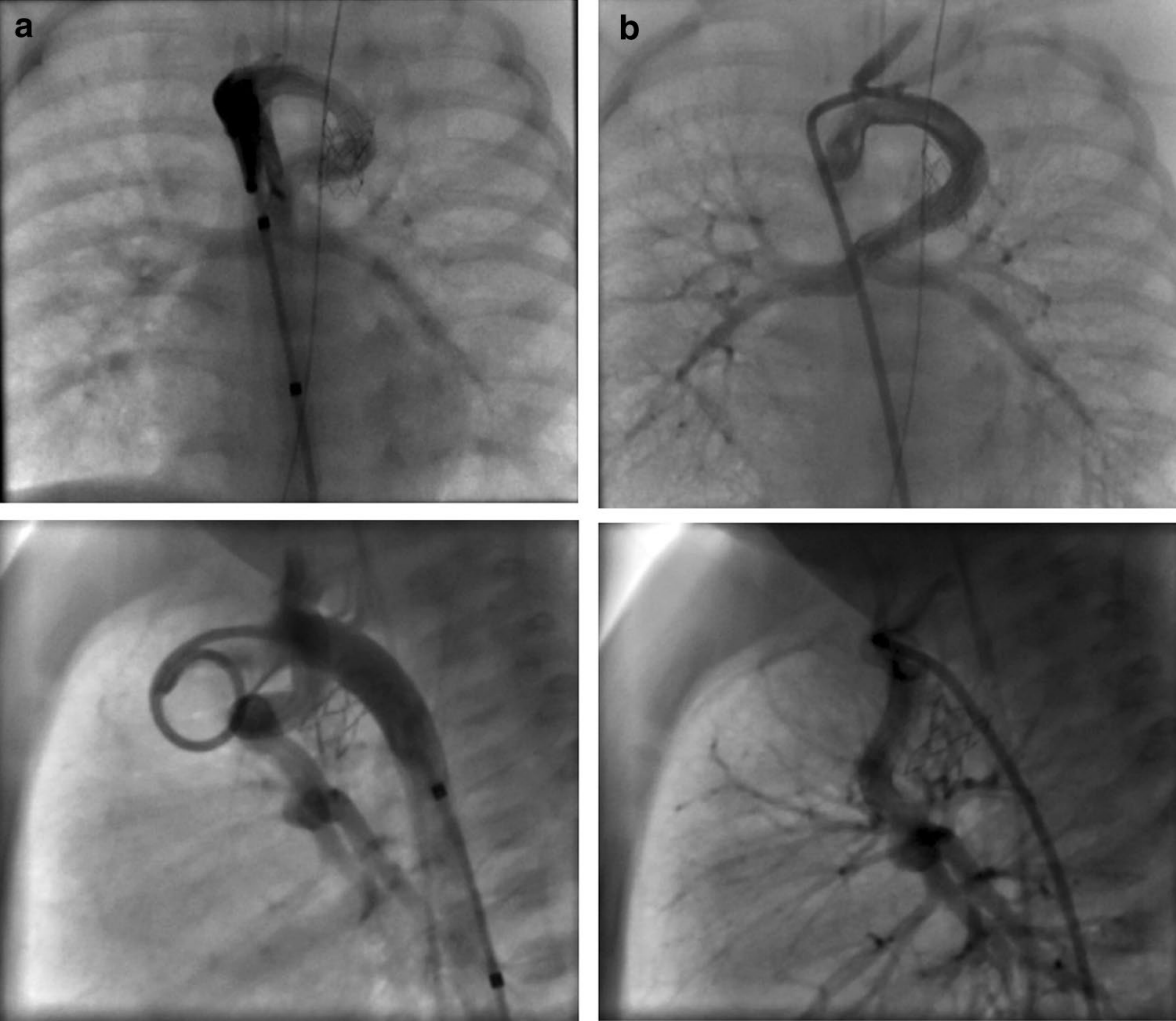
**a** Pre-PDA stent, **b** post-PDA stent

Despite medical treatment, the infant remained hypoxic and in distress. An obstructed flow pattern was noted in the vertical vein on echocardiogram. Cardiac catheterization results on DOL 2 confirmed pulmonary venous obstruction with a mean pressure gradient of 16 mm Hg across a discrete narrowing in the vertical vein (Fig. 1). She subsequently underwent placement of a 5 mm × 12 Palmaz-Blue stent in her vertical vein. Stenting of the PDA was delayed due to the acuity of the patient’s condition with significant pulmonary edema, and pulmonary hypertension which would have increased the risk of complications. On DOL 13, the patient underwent stenting of her PDA with two stents: Xience Alpine DES coronary stent (3.5 mm × 23 mm length) and Xience Alpine DES coronary stent (3.5 mm × 18 mm length) without complication (Fig. 2a, b).

At 2 months of age, she underwent repeat cardiac catheterization due to worsening hypoxia. This revealed a pressure gradient across the stented vertical vein of 12–13 mmHg. In preparation for a surgical TAPVR repair, she underwent balloon dilation of her PDA stents. This strategy was intended to allow the patient to undergo TAPVR repair, yet delay the necessity of the Glenn operation. The infant underwent repair of her supracardiac TAPVR with partial sutureless technique on DOL 70. She had an uneventful postoperative course, with echocardiographic evidence of unobstructed venous return.

At 5 months of age, she underwent a diagnostic catheterization prior to bidirectional Glenn. This catheterization showed a widely patent stented PDA with mean pulmonary artery pressures of 11–12 mmHg and confluent, well-developed branch pulmonary arteries. The TAPVR repair was unobstructed without evidence of pulmonary venous stenosis. The ostium primum atrial septal defect was unrestrictive, and the ventricular end-diastolic pressure was 7-8 mmHg. Based on these results, she was referred for bidirectional Glenn palliation.

At 6 months of age, our patient underwent a bidirectional Glenn, PDA division, and decellularized pulmonary homograft left pulmonary artery patch angioplasty (at the site of PDA stent extraction). Her postoperative course was uneventful and she was discharged home on POD 10. Subsequent echocardiograms have shown no Glenn anastomosis obstruction and unobstructed pulmonary venous return with trivial atrioventricular valve insufficiency.

## Discussion

Heterotaxy syndrome occurs in less than 2% of patients undergoing a congenital heart operation [3]. Associated anomalies include TAPVR, PA or PS, and, right or left atrial isomerism [1]. Additionally, SV anatomic variants, usually due to an unbalanced atrioventricular septal defect, are also seen and are more frequent in TAPVR patients with comorbid heterotaxy syndrome [4]. This subset of patients with heterotaxy, SV physiology, and TAPVR has historically had high early surgical mortality and poor long-term outcomes [4]. Patient-specific risk factors associated with higher mortality in this patient population include obstruction of pulmonary veins, atrioventricular valve regurgitation, distortion and/or discontinuity of the pulmonary arteries, and heart rhythm disturbances. Recognized procedure-specific risk factors include need for TAPVR repair at initial operation and younger age at time of initial surgery [5].

Transcatheter stent implantation to relieve obstructed pulmonary venous return offers the possibility of later surgical repair when the patient and cardiovascular structures are larger. Careful patient selection is important here, as the vertical vein stenosis must be safely approachable, and impingement on adjacent structures such as pulmonary arteries and bronchi with stent dilation must be considered. Our patient was amenable to vertical vein stenting as the stenosis was quite discrete, and remote from other important structures. The authors felt that vertical vein stenting here reduced the numerous demonstrable risks associated with neonatal surgical palliation in this complex subset of heterotaxy SV patients. Additionally, by stenting the PDA to support pulmonary blood flow, SV patients such as this with PA or PS may avoid the additive negative impact of surgical systemic-to-pulmonary artery shunting, and obviate the need for *any* surgical intervention in the neonatal period. Isolated vertical vein stenting has been described for high-risk neonates with obstructed TAPVR, and PDA stenting is increasingly supplanting surgical systemic-to-pulmonary artery shunt procedures [4, 5, 8]. We, however, found no previous reports which utilized stent implantation for both the initial treatment of obstructed TAPVR and PDA stabilization for sourcing of pulmonary blood flow.

## Conclusion

Heterotaxy syndrome includes various, unique thoracoabdominal sidedness relationships that increase the complexity of surgical intervention and management. Notably, patients with associated TAPVR and SV physiology pose an even greater challenge. Many of these patients undergo a first cardiac operation within 30 days of life [8]. The vast majority of these early initial cardiac operations include repair of TAPVR and have historically produced unsatisfactory outcomes. Transcatheter stenting of both the vertical vein and the PDA as an initial interventional strategy for selected patients with heterotaxy syndrome, obstructed TAPVR, and ductal-dependent pulmonary blood flow offers a unique strategy for early management. To the best of our knowledge, this is the first-reported case describing the successful use of balloon-expandable stents both to create unobstructed pulmonary venous blood flow and to maintain a source of systemic-to-pulmonary arterial blood flow. We speculate that this percutaneous interventional approach employed in the initial management of this infant was integral to the mitigation of her neonatal mortality risk.
